# Assessment of Equity in Access to Percutaneous Coronary Intervention (PCI) Centres in Poland

**DOI:** 10.3390/healthcare8020071

**Published:** 2020-03-26

**Authors:** Justyna Rój, Maciej Jankowiak

**Affiliations:** 1 Department of Operational Research, The Poznań University of Economics and Business, Al. Niepodległości 10, Poznań 61-875, Poland; 2 Department of Medical Law, Organisation and Healthcare Management, Poznań University of Medical Sciences, ul. Przybyszewskiego 39, Poznań 60-356, Poland; mjankowiak@ump.edu.pl

**Keywords:** equity, Gini coefficient, health care, invasive cardiology, Poland, regression

## Abstract

The purpose of this study is to analyse the disparities in the distribution of percutaneous coronary intervention (PCI) centres in Poland and the impact of eventual inequities on access to the invasive treatment of acute myocardial infarctions (AMI). To examine the distribution of PCI centres against population size and geographic size in Poland, the Gini coefficient calculated based on the Lorenz Curve was engaged. In addition, the regression function was employed to estimate the impact of distribution of PCI centres on access to invasive procedures (coronarographies and primary percutaneous coronary intervention). Data were collected from the public statistical system and Polish National Health Fund database for the year 2018. The relation and the level of equity was measured based on the aggregated data at a district (voivodeship) level. The results of the Gini coefficient analysis show that the distribution of invasive cardiology units measured against population size is more equitable than when measured against geographic size. In addition, the regression analysis shows the moderate size of the positive correlation between number of PCI centres per 100,000 population and the number of all categories of the invasive treatment of AMI per 100,000 population, and the lack of similar correlation in case of the number of PCI centres expressed per 1000 km^2^, which could be evidence of an insufficiency of PCI centres in areas where the concentration of PCI centres per 100,000 population is lower. The main implication for policy makers that results from this research is the need for a correction of PCI centres distribution per 100,000 inhabitants to ensure better access to invasive procedures.

## 1. Introduction

Diseases of the circulatory system are a frequent cause of mortality. Acute myocardial infarction (AMI) is one of these diseases, which would be fatal if not treated properly. Early invasive procedures (such as coronarography or especially primary percutaneous coronary intervention—primary PCI) can improve the outcomes of the treatment of AMI. Inequities in access to PCI and in survival after AMI according to socioeconomic factors were discussed in other studies [1,2]. There is a strictly defined maximal time to introduce the primary PCI procedure after myocardial infarction occurs. In the case of ST-segment elevation myocardial infarction (STEMI), time from the first medical contact (contact between a patient and a representative of a healthcare system) to the invasive treatment should be no longer than 120 min [3]. The same timing of the immediate invasive treatment is recommended for so-called very-high-risk myocardial infarction without persistent ST-segment elevation (NSTEMI) [4,5]. Excessive delay in the beginning of the invasive treatment reduces the advantages of primary PCI [6,7]. Therefore, there is a need for establishing a system of PCI centres in order to fulfil this treatment time recommendation. A regular geographical distribution of PCI centres could make time to the invasive treatment shorter. The European Society of Cardiology recommends organisation of the treatment of AMI in networks [3]. A network of PCI centres should be supported by an effective and rapid patient transportation system [8,9,10]. Outcomes of the myocardial infarction treatment could be improved by a shorter time to the implementation of invasive procedures in geographic areas, where a network of PCI centres is well developed [11]. Efficient functioning of the AMI invasive treatment system is based on easy access to PCI centres, including their adequate geographical distribution. Thus, this paper focuses on the equity in access to the invasive treatment (including primary PCI) of AMI in the context of geographical distribution of PCI centres (hospitals, where PCI procedures are performed). The aim of this study is the analysis of disparities in the distribution of PCI centres in Poland and the impact of eventual inequities on access to the invasive treatment of AMI.

As a research object, Poland was chosen for several reasons. Although cardiovascular diseases have been one of the main causes of death in the Polish population for years [12], the rates for the invasive cardiology services have been modified in order to make them more real terms beginning from 1 June 2016. Up to this moment, Poland has had satisfying outcomes of the AMI invasive therapy. As equitable access is an important value in all healthcare systems but also from the perspective of urgent cardiovascular incidents, therefore, it is of a high priority to verify the possible impact of such policy change. Thus, to the best knowledge of the authors, this is the first research on equity in the distribution of PCI centres (invasive cardiology infrastructure) in Poland of such a broad range. 

Poland has a general obligatory health insurance system [13]. In 1999, the system was completely decentralized [14] with 16 regional Health Insurance Organisations (HIOs) one in each region and one trade (nationwide) Health Insurance Organisation with a purchaser/payer function [15]. With the beginning of April 2003—HIOs have been replaced by the National Health Fund (NHF)—a single central insurance institution—with the Head Office of the NHF and 16 regional branches, one in each voivodeship in order to eliminate the regional differences in access to health care [16,17]. NHF is not only responsible for financing health services but also for establishing general rules for contracting as well as all processes of contracting [18]. 

Health and equity are important values in the Polish health care system. It arises not only from WHO constitutions and strategy [19] but also from Article 68 of the Polish Constitution [20]. One of the strategic objectives of the national health policy formulated in the National Health Programme [NHP] is the elimination of geographical and social inequalities in health. The key objectives for 2016 to 2020 are to extend the life expectancy of Poles, improve the quality of life related to health, and also to limit social inequities in health [21]. There is also a separate program applying to circulatory system diseases, which has the goal of reducing mortality due to heart and vascular diseases in Poland [22].

Despite the declining trend, still in 2014, cardiovascular diseases were responsible for 45.1% of all deaths in Poland, which is much higher than the average for the EU28 (38.1%) [23]. From the other side, the amount of public funds allocated by the NHF to finance cardiological services has been significantly increasing from approximately PLN 1 billion in 2004 to over PLN 3 billion in 2014. Moreover, the value of intervention services provided in acute coronary syndromes treatment has increased from PLN 0.2 billion to PLN 1.2 billion, and at the same time, there has been an increase from 19% to 39% of their share in the total value of cardiology services. In addition, the number of PCI centres has started to increase, especially non-public ones. Calculated per million inhabitants in 2014, there were more than four PCI centres in Poland, in a situation where according to the European guidelines, two such laboratories are sufficient for 24 hours [24]. However, the Supreme Audit Office report from the inspection of NHF branches showed that they did not carry out control of the quality and legitimacy of the services provided by invasive cardiology centres, even though such actions could minimise the risk of ineffective spending of NHF public funds. It could be also noticed that the activities of non-public PCI centres were focused mainly on invasive cardiology rather than non-invasive services [24]. Therefore, beginning from 1 June 2016, the rates for the invasive cardiology services have been modified in order to make them more real terms. It was also accompanied by a modification of the groups of cardiological services according to cardiologist suggestions. Until then, the outcomes of the AMI invasive therapy in Poland are satisfactory. According to OECD data, the 30-day mortality rate after admission to hospital following AMI in Poland in 2015 was lower than the EU average [25]. Thus, it is important to investigate the possible impact of these changes on the equity in Poland. 

## 2. Materials and Methods 

Data related to intervention cardiology units (PCI centres) used in this study were derived from the database of the Polish Association of Cardio-Vascular Interventions [26] for the year 2018. As Poland is divided into 16 districts (voivodeships), thus, this study used voivodeship-level data on inpatient health care providers, and each voivodeship was considered as a unit of analysis. The study data consisted of the number of PCI centres expressed as the number per 1000 km2 and per 100,000 people. Population and geographic area data were obtained from the Polish Statistical Yearbook 2018 [27]. Then, data related to the number of each type of cardiology intervention was derived from the Polish National Health Fund database [28] also for the year 2018. Table 1 contains research data divided into 16 voivodeships. There were 149 recognised PCI centres, which provide 24-hour service of urgent treatment of AMI. Most often, PCI centres usually are able to perform elective coronary interventions during office hours unless they are urgent procedures. On duty, the same PCI centres serve only urgent procedures. 

According to information published by the Polish NHF (Table 1) for each of the 16 Polish voivodeships, there were 70,307 hospitalisations due to the invasive treatment (coronarographies and PCIs together) of acute myocardial infarctions—AMI (ICD-10 categories: I21, I22, I23) in Poland in 2018. It includes 12,138 hospitalisations due to coronarographies and 58,169 hospitalisations due to PCI procedures. 

In 2018, there were 149 PCI centres serving urgent invasive procedures in the case of AMI in Poland (see Table 1). Table 2 shows that for PCI centres per 1000 km^2^, the mean value and its standard deviation in 2018 are 0.51 and 0.28. These two values demonstrate dispersion in the sample. Inversely, the number of PCI centres per 100,000 people shows less dispersion, as the mean value is 0.40 with a standard deviation of 0.10. 

The mean value of the number of hospitalisations per 100,000 population due to coronarographies and PCIs together was 186.87 with a standard deviation value of 37.28, while the mean value of those due to coronarographies was 33.41 with a standard deviation at 13.94, and that of those due to PCIs was 153.46 with a standard deviation of 25.07. The coefficient of variation (defined as the standard deviation divided by the mean value) for each of the above-mentioned three categories of invasive treatment of AMI was 19.9%, 41.7%, and 16.3%, respectively. That indicates a significantly stronger dispersion of the number of coronarographies compared with those of PCIs. The summary statistics of invasive treatment of myocardial infarctions are presented in Table 3. 

In order to examine the distribution of PCI centres against geographic size and population size in Poland, the Gini coefficient calculated based on the Lorenz curve was engaged. The Gini ratio is recognised as one of the most common measures of distribution and also as one of the superior tools for measuring inequity [29]. It was developed by the Italian Statistician Corrado Gini (1955) [30] as a summary measure of income inequality in society [31]. In this research, the Gini coefficient was calculated based on the Lorenz curve as a graphical representation [32,33]. As the Gini coefficient tends to be also defined as the ratio, thus, the numerator is the area between the Lorenz curve of the distribution and the uniform distribution line, and the denominator is the area under the uniform distribution line. Then, the actual extent of inequality is presented by the area between the Lorenz curve and the line of perfect equality. Less deviation from the line of perfect equality means more even distribution. The value of this ratio can range from 0 to 1, with 0 corresponding to perfect provider distribution (i.e., every region has this same number of providers per 100,000 people) and with 1, which means perfect provider inequality (i.e., one region has all the providers, while everyone else has zero of them). Then, the value of Gini coefficient below 0.3 means preferred equity status, from 0.3 to 0.4 means normal condition, while the Gini coefficient with the value between 0.4 and 0.6 triggers an alert of inequity, and the value exceeding 0.6 represents a highly inequitable state.

Two indicators were used for measuring inequity, reflecting the distribution of PCI centres—the first among populations and the second among geographical location—and the following formula was used:

*G(y)=∑^n^_i=1_ (2i-n-1)y_i_/n^2^y_mean_* (1)

where: 

y_i_ = value of i-observation

*n* = number of observations

In addition, the regression function was employed, as the parameters of the simple linear regression function were calculated in order to estimate the impact of the distribution of PCI centres on access to invasive procedures (coronarographies and PCIs) [34], which was given by the following general equation [35]:

Y = A_0_ + A_1_X (2)

where Y is a dependent variable, X is an independent variable, and A_0_ and A_1_ are the parameters of the linear regression equation. The independent variables (X) included the number of PCI centres per 1000 km^2^ and the number of PCI centres per 100,000 population. The dependent variables (Y) were the number of all invasive procedures (coronarographies and PCIs) performed in acute myocardial infarctions per 100,000 population, the number of PCIs performed in acute myocardial infarctions per 100,000 population, and the number of coronarographies performed in acute myocardial infarctions per 100,000 population.

The Pearson correlation coefficient was calculated for each of the regression functions as well in order to assess the size of correlation between dependent and independent variables. The formula given below was used for calculation of the Pearson correlation coefficient [35]:

r_X,Y_ = COV (X,Y) / D(X) D(Y) (3)

where r_X,Y_ is the Pearson correlation coefficient, COV (X,Y) is the covariance, D(X) is the standard deviation of the variable X, and D(Y) is the standard deviation of the variable Y. The size of correlation was evaluated as strong if r_X,Y_ was above 0.5, as moderate if r_X,Y_ was between 0.3 and 0.5, and as weak if r_X,Y_ was below 0.3. The parameters of linear regression functions and values of the Pearson correlation coefficient were calculated using STATISTICA software (TIBCO Software Inc., Statistica version 13. http://statistica.io. License number: JPZ711B316627AR-V).

## 3. Results

The Gini coefficient analysis doesn’t show significant inequalities in the distribution of PCI centres in Poland; however, it is more equitable if measured against population size (per 100,000 population) than against geographic size (per 1000 km^2^)—see Table 4.

One of the weaknesses of the Gini coefficient is namely that Lorenz curves can have different shapes, yet still yield similar Gini coefficients. Therefore, it is worth mentioning the shape of Lorenz curves. For both demographic and geographic dimensions, Lorenz curves are located under the equity curves. The Lorenz curve in the demographic dimension is similarly spaced from the equity curve, while the Lorenz curve in the geographic dimension coincides with the equality curve on 1/4 of its length, and then it is similarly spaced from it. 

**Figure 1:**
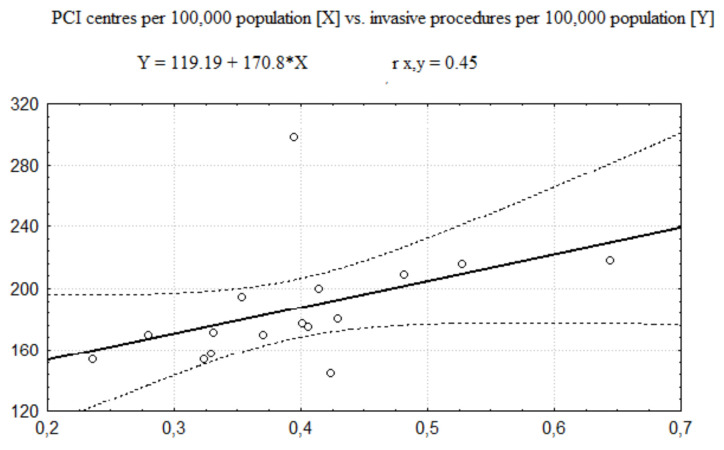
The regression function of PCI centres 100,000 population vs. number of invasive 100,000 population.

**Figure 2:**
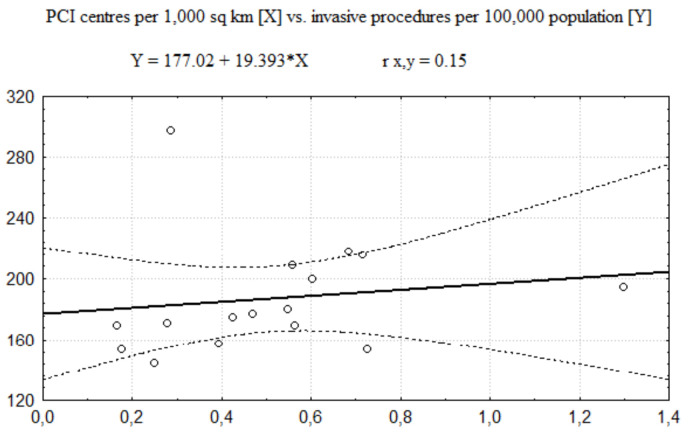
The regression function of PCI per centres per 1000 km^2^ vs. number of procedures per invasive procedures per 100,000 population.

The regression functions describing relationships between the number of PCI centres and the number of invasive procedures (coronarographies and PCIs together) performed in AMI is presented in Figure 1 (related to the number of PCI centres per 100,000 population) and Figure 2 (related to the number of PCI centres per 1000 km^2^). In these cases, the Pearson correlation coefficient values (see Table 5) show a moderate size of positive correlation between the number of invasive procedures and the number of PCI centres per 100,000 population and no significant correlation between the number of invasive procedures and the number of PCI centres per 1000 km^2^.

**Figure 3:**
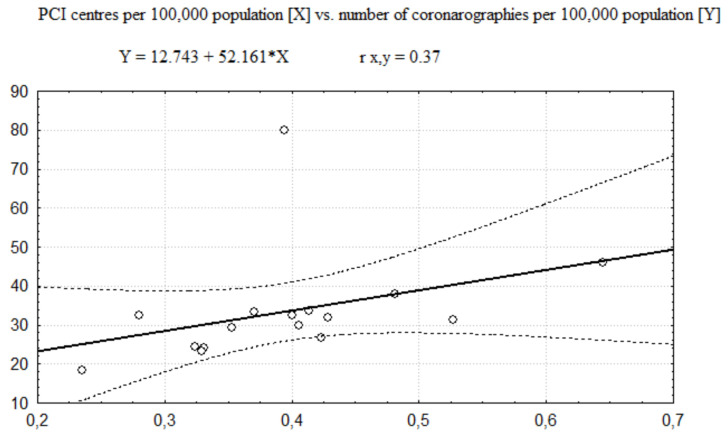
The regression function of PCI centres per 100,000 population vs. number of coronographies per 100,000 population.

**Figure 4:**
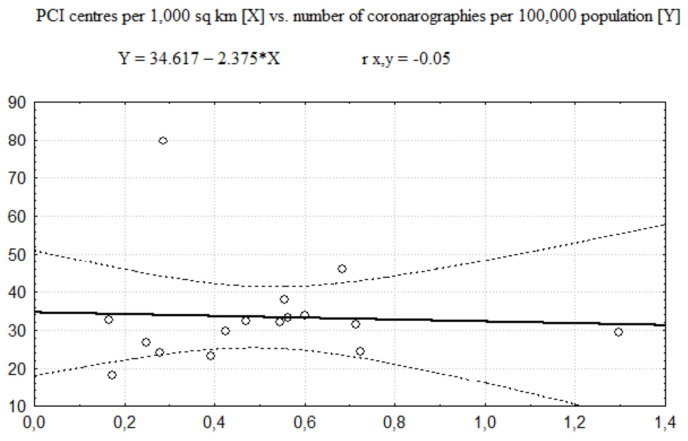
The regression function of PCI centres per 1000 km^2^ vs. number of coronographies per 100,000 population.

In further analysis, the category of invasive procedures, consisting of coronarographies and PCIs together, was decomposed. The regression functions between the number of PCI centres and the number of coronarography-only procedures are presented in Figure 3 and Figure 4 (respectively showing regression for the number of PCI centres expressed per 100,000 population and per 1000 km^2^). The Pearson correlation coefficient values (see Table 5) show that size of positive correlation between the number of coronarographies and the number of PCI centres per 100,000 population is still moderate although weaker than in the case of invasive procedures altogether. However, there is no distinct correlation between the number of coronarographies and the number of PCI centres per 1000 km^2^ as well.

**Figure 5:**
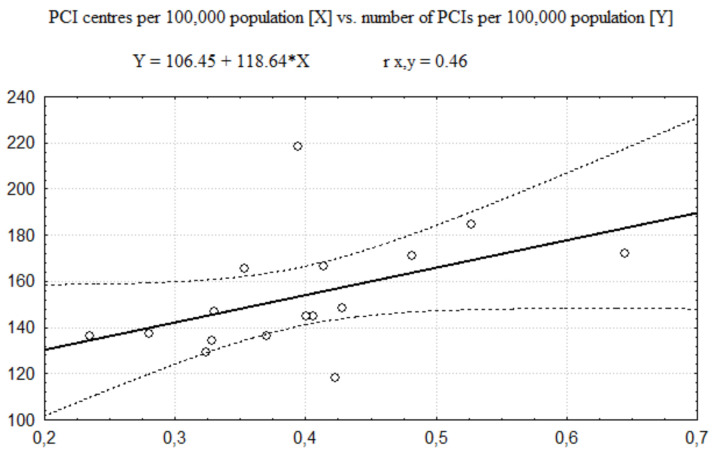
The regression function of PCI centres per 100,000 population vs. number of PCIs 100,000 population.

**Figure 6:**
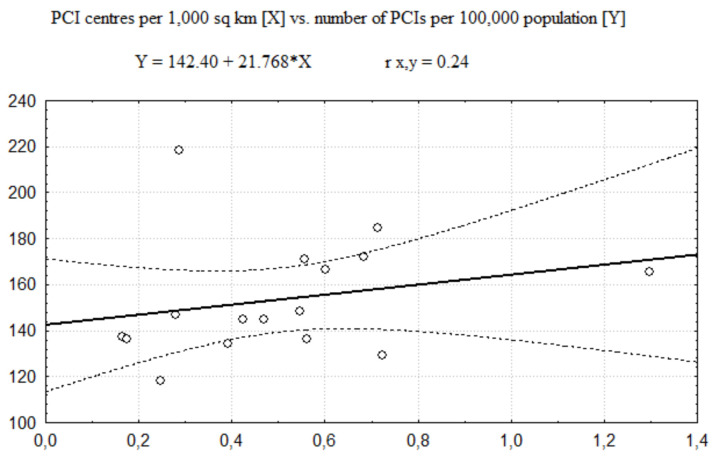
The regression function of PCI centres number per 1000 km^2^ vs. number of PCIs per 100,000 population.

Finally, the regression between the number of PCI centres and the number of PCIs only is presented in Figure 5 (related to the number of PCI centres per 100,000 population) and Figure 6 (related to the number of PCI centres per 1000 km^2^). The values of the Pearson correlation coefficient (presented in Table 5) suggest that size of positive correlation between the number of PCIs and the number of PCI centres per 100,000 population is moderate as well (stronger than in the previous two categories); meanwhile, there is no significant correlation in the case of PCI centres per 1000 km^2^, which is similar to the previously described two categories of the AMI invasive treatment.

Recapitulating, the results of this research show a moderate size of positive correlation between the number of PCI centres per 100,000 population and the number of all categories of the invasive treatment of AMI per 100,000 population, and lack of similar correlation in case of the number of PCI centres expressed per 1000 km^2^. Values of the Pearson correlation coefficient for all considered categories were collected together in Table 5.

## 4. Discussion

This research is focused on the identification of inequalities in access to the invasive treatment of AMI in Poland. Inequalities in the treatment of coronary heart disease and acute myocardial infarctions due to socioeconomic factors (such as age, economic status, level of education, etc.) in other countries were indicated in earlier research [1,2,36,37]. Some other studies were conducted about a relationship between the volume of PCI performed in hospitals and patient outcomes [38,39].

Disparities in medical management can emerge from differences in the infrastructure of health care systems as well [40,41]. The infrastructure of healthcare consists of, among other things, buildings, medical equipment, and medical professionals. In case of the invasive treatment of AMI, the infrastructure facilities are available in PCI centres. The harmonious distribution of invasive cardiology units is conducive to a reduction of inequalities in the AMI treatment. In this research, the impact of disparities in the distribution of PCI centres on access to the invasive treatment of AMI in Polish population was examined.

The distribution of PCI centres against in both population size and geographical size—based on the Gini coefficients—appeared to be quite equitable, which means that the implementation of changes (in July 2016) in the price and structure of invasive cardiology services did not cause the inequity in the distribution of PCI centres either by 100,000 population or by 1000 km^2^. However, a lower level of equity in the case of geographical distribution could be the result of the sparsity of the population. Probably, most of the Polish PCI centres are distributed within the developed districts and especially in large cities of developing districts. However, this problem would require further analysis, which should be expanded by including for example the analysis of the morbidity on acute myocardial infarction. However, the access to such data is limited because such data are not collected according to the district’s level. 

In the model-based situation (no limits on the side of health care infrastructure), the number of existing PCI centres shouldn’t affect access to the invasive treatment of AMI, and recruitment to coronarography or PCI should be based only on medical guidelines. On the assumption of an equal incidence of AMI in each of research units and Polish voivodeships (presupposition perhaps theoretical, but useful for simplification of the model), any shortage in the number of PCI centres in relation to medical needs could appear as a dependence of the number of invasive procedures on the distribution of PCI centres. On the other hand, a lack of such dependence could be interpreted as an indicator of sufficient accessibility to the invasive treatment.

In fact, the analysis of regression between the number of PCI centres, expressed per 1000 km^2^, and the number of all categories of invasive procedures (coronarographies and PCIs) implemented in AMI shows no significant correlation. The range of the Person correlation coefficient was between −0.05 and 0.24, which indicates no substantial dependence of the invasive treatment of AMI on the degree of PCI centres concentration per 1000 km^2^. According to these findings, the geographical distribution of PCI centres (measured as the number of PCI centres per 1000 km^2^ of each research unit—a voivodeship) couldn’t be perceived as a barrier to the invasive treatment of AMI. In that geographical respect, there can’t be confirmed substantial inequalities in access to the invasive management in AMI among districts (voivodeships) of Poland. These findings are consistent with the outcomes of the Gini coefficient analysis where the value of the Gini coefficient was about 28%, not indicating substantial inequities in the distribution of invasive cardiology units per 1000 km^2^. 

Otherwise, the analysis of regression between the number of PCI centres, expressed per 100,000 Polish population, and the number of all invasive procedures categories (coronarographies and PCIs) implemented in AMI shows a moderate size of correlation. The Person correlation coefficient was 0.37 for coronarographies, 0.46 for PCIs, and 0.45 for altogether invasive procedures. On the assumption of a similar incidence of AMI in each of the Polish voivodeships, it indicates dependence of the invasive treatment of AMI on the degree of concentration of PCI centres per 100,000 population. This kind of dependence could be evidence of a shortage of PCI centres in areas where the concentration of PCI centres per 100,000 population is lower, suggesting that access to the invasive treatment in these areas is limited by healthcare infrastructure. It is likely that an increase of the number of PCI centres in these areas could improve management in AMI. This result of regression doesn’t confirm the outcomes of the Gini coefficient analysis, which have a value of about 13%; this doesn’t indicate significant inequalities in the distribution of invasive cardiology units per 100,000 population. The possible explanation of a discrepancy between outcomes of the Gini coefficient analysis and the regression analysis could be considerable differences in the distribution of PCI centres per 100,000 inhabitants at lower than a district level. Another factor that could influence the number of invasive procedures is an imbalance of the AMI incidence among examined districts.

While the number of PCI centres per 100,000 population is positively correlated with the number of cardiology invasive services performed, the main implication for policy makers is the necessity of correction of PCI centres distribution per 100,000 to ensure better access to invasive procedures. This action is needed in the areas (at a district level or lower) where the amount of invasive cardiology units per capita is relatively low. In addition, policy makers should create a more detailed database. 

The lack of inclusion of an AMI incidence parameter could be perceived as the important limitation of this model, but on the other hand, it is based only on existing data derived by NHF. Particularly, the incorporation of detailed information on the actual incidence of AMI in examined units into the regression model should be essential for its better validity and prediction ability. Especially, there are some differences in AMI incidence between voivodeships in Poland. 

Further research with the usage of more detailed data on the incidence and the invasive treatment of AMI (which is presently not available for lower than a district level of the territorial division) is reasonable in order to precisely identify the needs within invasive cardiology infrastructure. It is of high importance, as there are only studies on the coronary heart disease (CHD)/AMI mortality in Poland [42,43,44]. The direction of future research could be also the investigation of socioeconomic factors (such as age, economic status, level of education, urban/rural inhabitancy, etc), which are mentioned in the literature for other countries [36,37,38,39] as well as in an EC report [45], and which can influence the equity in the treatment of coronary heart disease and acute myocardial infarction. The voivodeships in Poland especially differ in these parameters [46,47]; however, it would require more detailed databases regarding the CHD as well as AMI incidence, etc. 

## 5. Conclusions

On the basis of Gini coefficients, the distribution of invasive cardiology units against both population size and geographical size appears to be balanced; however, it is more equitable if measured against population size than against geographic size. Based on the regression model, our research shows no significant dependence between the number of PCI centres per 1000 km^2^ and the number of invasive procedures conducted in the treatment of AMI. It can indicate that the geographical distribution of invasive cardiology units isn’t a barrier to access the invasive procedures in AMI in Poland.

Otherwise, regression indicates a substantial dependence of the invasive treatment of AMI on the number of PCI centres per 100,000 population. It could lead to inequalities in access to the invasive treatment of AMI and restrictions in the areas where the number of PCI centres per 100,000 population is lower. An increase in the number of invasive cardiology labs in these worse-equipped areas of Poland could improve the treatment of AMI. The control of the quality and legitimacy of the services provided by PCI centres, carried out by NHF, would be helpful. 

Thus, the results of this study need to be taken into account by policy makers when formulating the complex actions, rather than increasing simply the number of PCIs. In practice, this study can be used as a basis for healthcare policy formulation in order to correct the unequal distribution of PCI. By having such information, national government could monitor the nationwide distribution of PCI and provide some advice to regional policy makers (including NHF) in order to make proper adjustments. This would allow balancing PCI in different geographical areas. 

The collection of more detailed statistical data (related to the incidence and the invasive treatment of AMI on a district level and lower) by health care authorities could be useful for further research and development of the regression model with the aim of the better identification of the needs within invasive cardiology infrastructure. Thus, it would be possible to include more epidemiological and demographic differences between regions and macro regions. 

## Figures and Tables

**Table 1:** Characteristics of Polish districts (voivodeships) as units of analysis.

District (Voivodeship)	Area in Squar km ^a^	Population ^a^	Number of PCI Centres ^b^	Hospit. due to Coronarographies ^c^	Hospit. due to PCI Procedures ^c^
Lower Silesian	19,947	2,901,225	12	978	4828
Kuyavian-Pomeranian	17,972	2,077,775	10	786	3557
Lubelskie	25,122	2,117,619	7	512	3115
Lubuskie	13,988	1,014,548	4	810	2216
Łódź	18,219	2,466,322	13	773	4556
Lesser Poland	15,183	3,400,577	11	830	4402
Masovian	35,558	5,403,412	20	1794	7373
Opole	9412	986,506	4	294	1430
Podkarpackie	17,846	2,129,015	7	493	2860
Podlaskie	20,187	1,181,533	5	316	1394
Pomeranian	18,310	2,333,523	10	745	3465
Silesian	12,333	4,533,565	16	1331	7498
Świętokrzyskie	11,711	1,241,546	8	570	2136
Warmian-Masurian	24,173	1,428,983	4	465	1965
Wielkopolska	29,826	3,493,969	14	1130	5060
Zachodniopomorskie	22,892	1,701,030	4	311	2314
Total - Poland	312,679	38,411,148	149	12,138	58,17

Source: ^a^ Statistics Poland (Główny Urząd Statystyczny) 2018. https://stat.gov.pl; ^b^ Database of Association of Cardiovascular Interventions 2018. http://www.aisn.pl; ^c^ Database of Polish National Health Fund 2018. https://statystyki.nfz.gov.pl.

**Table 2:** Summary statistics of percutaneous coronary intervention (PCI) centres in Poland (2018).

PCI Centres	Mean	Median	Max.	Min.	Std. Dev. *	Variance
Per 1000 km^2^	0.51	0.51	1.30	0.17	0.28	0.08
Per 100000 people	0.40	0.40	0.64	0.23	0.10	0.01

Source: Authors’ calculation based on data by AISN: http://www.aisn.pl/pracownie/baza_pracowni * Standard deviation.

**Table 3:** Summary statistics of the invasive treatment of acute myocardial infarctions (AMI) in Poland (2018).

Statistics	Hospitalisations per 100,000 Population due to:		
	Coronarographies and PCIs	Coronarographies Only	PCIs Only
Mean value	186.87	33.41	153.46
Median	175.96	31.63	146.03
Maximum value	298.26	79.84	218.42
Minimum value	144.73	18.28	117.98
Standard deviation	37.28	13.94	25.07
Coefficient of variation	19.9%	41.7%	16.3%

Source: Authors’ calculation based on data by the Polish National Health Fund: https://statystyki.nfz.gov.pl.

**Table 4:** Gini coefficient in 2018.

PCI Centres	Gini Coefficient
Per 1000 km^2^	28.02%
Per 100,000 population	12.87%

Source: Authors’ calculation.

**Table 5:** Values of Person correlation coefficient (r_X,Y_) for the relationship between the number of PCI centres and number of invasive procedures in the treatment of AMI.

Number of PCI Centres:	Hospitalisations Per 100,000 Population due to:		
	Coronarographies and PCIs	Coronarographies Only	PCIs Only
Per 100,000 population	r_X,Y_ = 0.45	r_X,Y_ = 0.37	r_X,Y_ = 0.46
Per 1000 km^2^	r_X,Y_ = 0.15	r_X,Y_ = −0.05	r_X,Y_ = 0.24

Source: Authors’ calculation using STATISTICA software.
